# Characterizing Risk of In-Hospital Mortality Following Subarachnoid Hemorrhage Using Machine Learning: A Retrospective Study

**DOI:** 10.3389/fsurg.2022.891984

**Published:** 2022-06-08

**Authors:** Jiewen Deng, Zhaohui He

**Affiliations:** Department of Neurosugery, The First Affiliated Hospital of Chongqing Medical University, Chongqing, China

**Keywords:** machine learning, SAH, prediction model, recursive feature abstraction, subarachnoid hemorrhage

## Abstract

**Background:**

Subarachnoid hemorrhage has a high rate of disability and mortality, and the ability to use existing disease severity scores to estimate the risk of adverse outcomes is limited. Collect relevant information of patients during hospitalization to develop more accurate risk prediction models, using logistic regression (LR) and machine learning (ML) technologies, combined with biochemical information.

**Methods:**

Patient-level data were extracted from MIMIC-IV data. The primary outcome was in-hospital mortality. The models were trained and tested on a data set (ratio 70:30) including age and key past medical history. The recursive feature elimination (RFE) algorithm was used to screen the characteristic variables; then, the ML algorithm was used to analyze and establish the prediction model, and the validation set was used to further verify the effectiveness of the model.

**Result:**

Of the 1,787 patients included in the mimic database, a total of 379 died during hospitalization. Recursive feature abstraction (RFE) selected 20 variables. After simplification, we determined 10 features, including the Glasgow coma score (GCS), glucose, sodium, chloride, SPO_2_, bicarbonate, temperature, white blood cell (WBC), heparin use, and sepsis-related organ failure assessment (SOFA) score. The validation set and Delong test showed that the simplified RF model has a high AUC of 0.949, which is not significantly different from the best model. Furthermore, in the DCA curve, the simplified GBM model has relatively higher net benefits. In the subgroup analysis of non-traumatic subarachnoid hemorrhage, the simplified GBM model has a high AUC of 0.955 and relatively higher net benefits.

**Conclusions:**

ML approaches significantly enhance predictive discrimination for mortality following subarachnoid hemorrhage compared to existing illness severity scores and LR. The discriminative ability of these ML models requires validation in external cohorts to establish generalizability.

## Introduction

Subarachnoid hemorrhage (SAH) is a type of hemorrhagic stroke that accounts for 3% of all stroke types. With the development of medicine, the global case fatality rate has decreased from 50% to 17%, but the mortality rate of subarachnoid hemorrhage remains high (1–3). In addition, survivors are often left with a permanent disability, cognitive deficits (particularly in executive function and short-term memory), and mental health symptoms (depression, anxiety), leading to significant reductions in health-related quality of life. In recent years, machine learning (ML), as an area of artificial intelligence, has been able to learn from data based on computational modeling. Similarly, ML can fit higher-order relationships between covariates and outcomes in data-rich environments (4–6).

The purpose of this study was to determine whether ML algorithms using demographics, comorbidities, laboratory tests, and other variables can predict the prognosis of SAH fairly accurately and to identify factors that contribute to predictive ability.

## Patient Selection and Methods

### Data Source

This study was a retrospective study based on the Medical Information Mart for Intensive Care IV (7) (MIMIC-IV version 1.0) database. An individual who has finished the Collaborative Institutional Training Initiative examination (Certification number 43357625 for author Deng) can access the database.

### Participant Selection

Inclusion criteria are as follows: (1) patients with subarachnoid hemorrhage confirmed by ICD-9 or ICD-10; (2) people with an age of more than 16 years old; and (3) admission to ICU with the Glasgow coma score (GCS). Moreover, for patients with ICU admissions more than once, only data of the first ICU admission of the first hospitalization were included in the analysis.

### Predictors

In this study, the data were extracted from MIMIC-IV, including age, gender, race, language, GCS, sepsis-related organ failure assessment (SOFA) score, and history of trauma. Then, we extracted data containing vital signs, laboratory findings, treatment history of heparin, and antibiotics during hospitalization. Besides, we collected the Charlson comorbidity index (CCI) composed of myocardial infarction, congestive heart failure, peripheral vascular disease, cerebrovascular disease, dementia, chronic pulmonary disease, rheumatic disease, peptic ulcer disease, diabetes, paraplegia, renal disease, malignant cancer, severe liver disease, metastatic solid tumor, and acquired immunodeficiency syndrome (AIDS).

### Outcomes

Patients diagnosed with subarachnoid hemorrhage died during hospitalization.

### Statistical Analysis

Categorical variables were presented as numbers and percentages that were analyzed using the *χ*^2^ test or the Fisher exact test, while continuous variables were expressed as mean ± SD or median with interquartile range (IQR), which were analyzed by an independent *t*-test or Mann–Whitney *U* test.

Each feature has different importance or coef attributes in the model, and these data determine the importance of the feature in the model. Recursive feature elimination (RFE) returns the importance of each feature through the learner (8, 9). Then, the least important feature is removed from the current feature set. This step of recursion on the feature set is repeated until the required number of features is finally reached. Then, features are then considered in groups of 5–60; they are organized according to the grade obtained by the feature selection method. In order to find the best hyperparameters, 10-fold cross-validation is used as a resampling method. In each iteration, every nine folds are used as a training subset, and the remaining one is processed to adjust the hyperparameters. In this way, each sample will participate in the training model and test the model, so that all data can be used to the greatest extent.

In this study, we divided the data set (ratio 70:30), trained the model, and verified it. We calculated the median and 95% confidence interval of the area under the curve (AUC), where the AUC value of 1.0 indicated complete discrimination and 0.5 indicated no discrimination. Finally, the accuracy, sensitivity, specificity, negative predictive value, and positive predictive value of external data verification were calculated. Additionally, we conducted the decision curve analysis (DCA) to determine the clinical usefulness of the included variables by quantifying the net benefit at different threshold probabilities.

All analyses were performed by the statistical software package R version 4.1.3 (http://www.R-project.org, The R Foundation). In our study, we used the “Caret” R packages to achieve the process. *P* values less than 0.05 (two-sided test) were considered statistically significant.

## Results

### Baseline Characteristics

**Figure 1:**
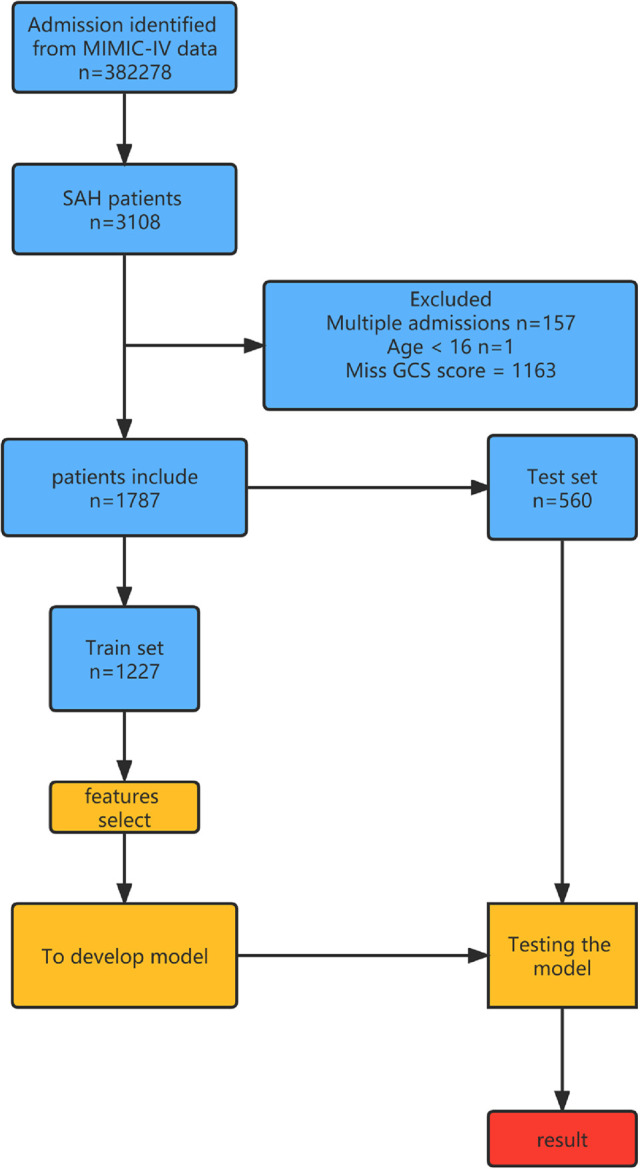
Overview of the methods used for data extraction, training, and testing. MIMIC-IV, Medical Information Mart for Intensive Care-IV; SAH, subarachnoid hemorrhage.

Variable values of the SAH patients in MIMIC-IV were analyzed. A total of 1,787 cases were included in the study, of which 349 died during hospitalization. It is found from the data in the table that the infection indexes of the dead patients are significantly increased, and the coagulation system has an abnormal function, thrombocytopenia, electrolyte disorder, and so on. At the same time, the temperature and oxygen saturation of these patients fluctuate more widely and are more likely to be accompanied by other diseases (Table 1 and Figure 1).

**Table 1:** Baseline characteristics of MIMIC-IV.

	Survival (*n* = 1,438)	Dead in hospital (*n* = 349)	*P*-value
Baseline characteristics			
Age (year)	63 (51, 76)	70 (59, 82)	<0.001
Sex (female)	720 (50.07%)	169 (48.42%)	0.581
Race			
Black	91 (6.33%)	17 (4.87%)	<0.001
White	932 (64.81%)	163 (46.70%)	
Hispanic	65 (4.52%)	10 (2.87%)	
Asian	46 (3.20%)	13 (2.87%)	
Others	304 (21.14%)	136 (38.97%)	
Language			
English	1,283 (89.22%)	155 (44.41%)	0.714
Unknow	309 (21.49%)	40 (11.46%)	
Marital			
Single	368 (25.59%)	48 (13.75%)	<0.001
Married	629 (43.74%)	120 (34.38%)	
Divorced	98 (6.82%)	18 (5.16%)	
Widowed	159 (11.06%)	43 (12.32%)	
Unknow	184 (12.80%)	120 (34.38%)	
Weight	75.50 (64.00, 88.00)	73.00 (61.70, 87.30)	0.033
Trauma	697 (48.47%)	109 (31.23%)	<0.001
Coexisting disorders			
Myocardial infarction	109 (7.58%)	42 (12.03%)	0.007
Congestive heart failure	148 (10.29%)	53 (15.19%)	0.009
Peripheral vascular disease	87 (6.05%)	26 (7.45%)	0.335
Cerebrovascular disease	815 (56.68%)	255 (73.07%)	<0.001
Dementia	62 (4.31%)	13 (3.72%)	0.624
Chronic pulmonary disease	187 (13.00%)	55 (15.76%)	0.177
Rheumatic disease	26 (1.81%)	6 (1.72%)	0.911
Peptic ulcer disease	8 (0.56%)	3 (0.86%)	0.516
Diabetes	250 (17.39%)	75 (21.49%)	0.075
Paraplegia	150 (10.43%)	49 (14.04%)	0.055
Renal disease	118 (8.21%)	51 (14.04%)	<0.001
Malignant cancer	51 (3.55%)	20 (5.73%)	0.061
Severe liver disease	19 (1.32%)	12 (3.44%)	0.007
Metastatic solid tumor	18 (1.25%)	9 (2.58%)	0.068
AIDS	3 (0.21%)	2 (0.57%)	0.248
Vital signs (1st 24 h)			
Heart rate (min)	77.49 (69.64, 87.88)	80.89 (73.26, 93.58)	<0.001
Temperature (°C)	37.00 (36.78, 37.24)	37.00 (36.51, 37.45)	0.001
SBP (mmHg)	124 (115, 134)	123 (111, 134)	<0.001
DBP (mmHg)	65 (58, 72)	62 (55, 70)	<0.001
MBP (mmHg)	82 (75, 88)	80 (73, 88)	<0.001
Respiratory rate (min)	18 (16, 20)	19 (17, 22)	<0.001
SPO_2_	97.28 (96.00, 98.72)	97.88 (95.55, 99.25)	<0.001
Laboratory			
WBC	9.78 (7.91, 11.78)	12.59 (10.27, 15.70)	<0.001
Hematocrit	33.86 (29.80, 37.70)	32.93 (28.55, 36.90)	0.017
Hemoglobin	11.23 (9.86, 12.69)	10.85 (9.21, 12.33)	0.001
Mch	30.58 (29.40, 31.81)	30.55 (29.30, 31.86)	0.492
Mchc	33.31 (32.47, 34.15)	33.07 (32.05, 33.87)	<0.001
Mcv	91.00 (88.00, 95.00)	92.00 (88.00, 96.00)	0.039
RBC	3.70 (3.25, 4.15)	3.52 (3.10, 4.10)	0.008
Rdw	13.72 (13.03, 14.79)	14.48 (13.50, 15.95)	<0.001
Platelet	228.68 (183.00, 287.00)	194.86 (140.00, 249.33)	<0.001
Neutrophils	77.67 (73.30, 83.95)	78.50 (77.58, 86.00)	0.008
Lymphocytes	13.94 (9.20, 16.80)	12.50 (6.70, 14.20)	<0.001
Monocytes	5.87 (4.20, 7.00)	5.87 (4.33, 6.23)	0.482
Eosinophils	0.90 (0.30, 1.30)	0.75 (0.20, 1.13)	0.002
Basophils	0.35 (0.20, 0.45)	0.30 (0.20, 0.36)	<0.001
Bicarbonate	24.48 (22.90, 26.00)	23.25 (20.00, 24.62)	<0.001
Bun	14.50 (11.00, 19.50)	20.00 (14.67, 30.25)	<0.001
Calcium	8.64 (8.35, 8.95)	8.45 (8.08, 8.66)	<0.001
Chloride	103.90 (101.60, 106.00)	105.67 (103.00, 111.20)	<0.001
Creatinine	0.75 (0.61, 0.93)	0.95 (0.70, 1.40)	<0.001
Glucose	119.23 (107.00, 136.00)	150.65 (131.38, 178.10)	<0.001
Sodium	139.35 (137.44, 141.50)	141.00 (139.00, 145.19)	<0.001
Potassium	3.93 (3.75, 4.18)	4.00 (3.80, 4.32)	<0.001
INR	1.12 (1.05, 1.22)	1.20 (1.10, 1.40)	<0.001
PT	12.38 (11.57, 13.50)	13.34 (12.10, 15.10)	<0.001
APTT	28.28 (25.95, 30.93)	29.00 (26.20, 34.31)	<0.001
NLR	5.57 (4.42, 9.06)	6.40 (5.46, 12.60)	<0.001
Therapy			
Heparin	1,154 (80.25%)	157 (44.99%)	<0.001
Antibiotic	823 (57.23%)	226 (64.76%)	0.010
Scoring system			
SOFA	3 (2, 5)	6 (4, 8)	<0.001
GCS	13 (10, 14)	9 (3, 15)	<0.001

*MIMIC-IV, Medical Information Mart for Intensive Care-IV; AIDS, acquired immunodeficiency syndrome; SBP, systolic blood pressure; DBP, diastolic blood pressure; MBP, mean blood pressure; Mch, mean corpuscular hemoglobin; Mchc, mean corpuscular hemoglobin concentration; Mcv, mean corpuscular volume; RBC, red blood cell; Rdw, red blood cell volume distribution width; SPO_2_, oxygen saturation; GCS, Glasgow coma score; SOFA, sepsis-related organ failure assessment; NLR, neutrophil-to-lymphocyte ratio.*

### Variable Importance

**Figure 2:**
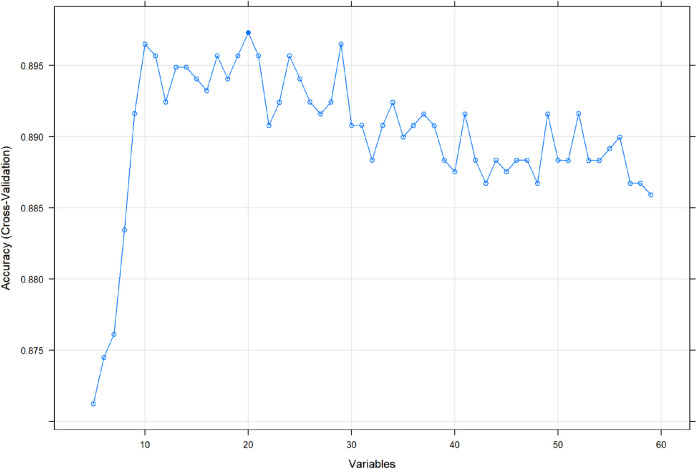
Correlation diagram between different feature numbers and accuracy in the RFE algorithm. RFE, recursive feature elimination.

Through feature screening by the RFE algorithm, we find that it has the highest accuracy when 20 features are included (Figure 2). In order to further simplify the model, we choose the models with an accuracy similar to the best feature number to verify the analysis. Therefore, we establish the prediction model with the characteristic numbers of 10 and 20. Model1 includes GCS, glucose, sodium, chloride, SPO_2_, bicarbonate, temperature, white blood cell (WBC), heparin use, and SOFA score, while Model2 include GCS, glucose, sodium, chloride, SPO_2_, bicarbonate, temperature, white blood cell (WBC), heparin use, SOFA score, creatinine, bun, platelet, age, marital, trauma, lymphocytes, calcium, race, and cerebrovascular disease. Then, these variables were used in all the subsequent analyses for all models in both training and testing sets.

### Prediction performance of different models

**Figure 3:**
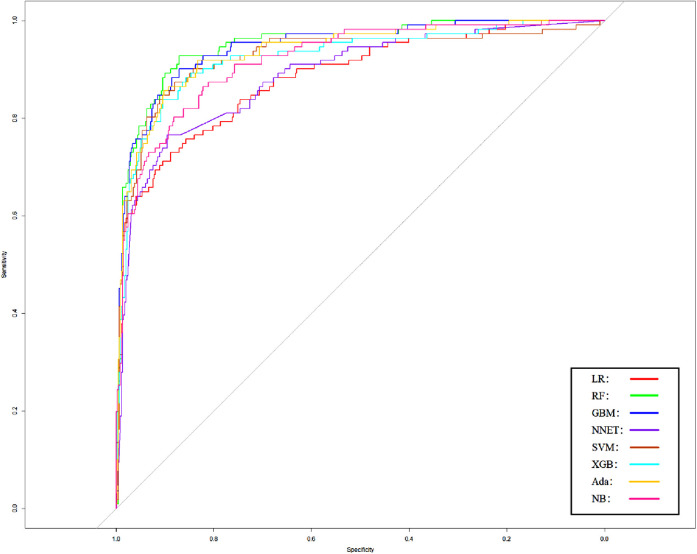
Area under receiver operating characteristic curve by different Model1 algorithms in the validation cohort. LR, logistic regression; RF, random forest; GBM, gradient boosting machine; NNET, artificial neural network; SVM, support vector machine; XGB, eXtreme gradient boosting; Ada, adapting boosting; NB, naïve Bayes; Model1 was adjusted for GCS, glucose, sodium, chloride, SPO_2_, bicarbonate, temperature, white blood cell (WBC), heparin use, and SOFA score.

**Figure 4:**
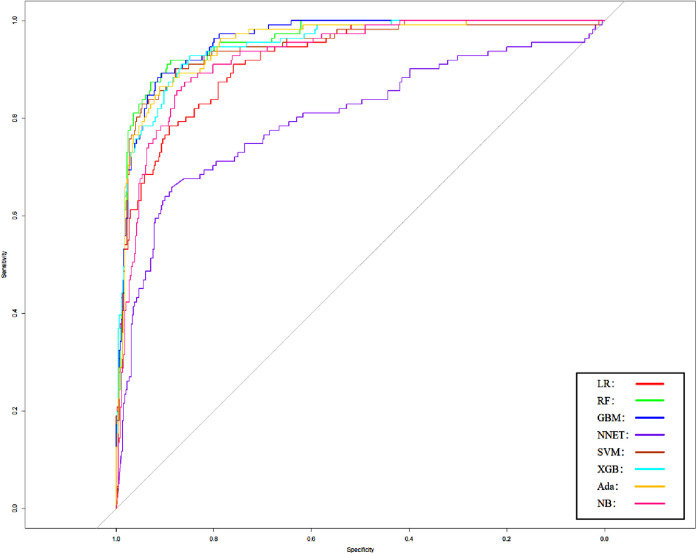
Area under the receiver operating characteristic curve by different Model2 algorithms in the validation cohort. LR, logistic regression; RF, random forest; GBM, gradient boosting machine; NNET, artificial neural network; SVM, support vector machines; XGB, eXtreme gradient boosting; Ada, adapting boosting; NB, naïve Bayes; Model2 was adjusted for GCS, glucose, sodium, chloride, SPO_2_, bicarbonate, temperature, white blood cell (WBC), heparin use, SOFA score, creatinine, bun, platelet, age, marital, trauma, lymphocytes, calcium, race, and cerebrovascular disease.

**Figure 5:**
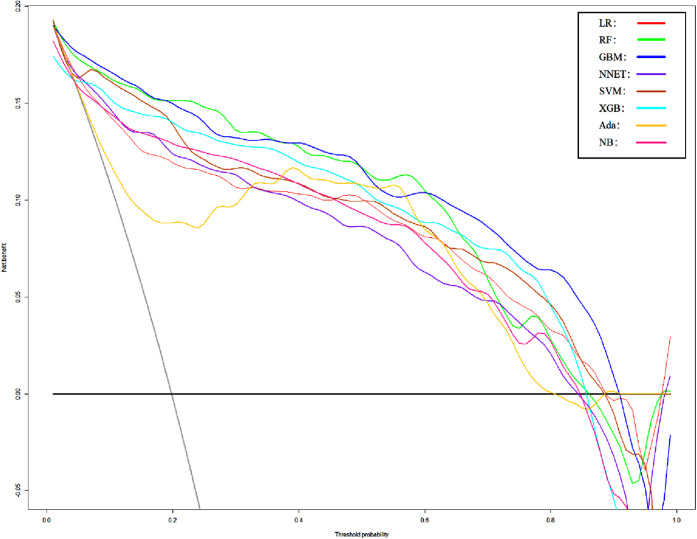
Decision curve analysis of Model1. LR, logistic regression; RF, random forest; GBM, gradient boosting machine; NNET, artificial neural network; SVM, support vector machine; XGB, eXtreme gradient boosting; Ada, adapting boosting; NB, naïve Bayes; Model1 was adjusted for GCS, glucose, sodium, chloride, SPO_2_, bicarbonate, temperature, white blood cell (WBC), heparin use, and SOFA score.

**Figure 6:**
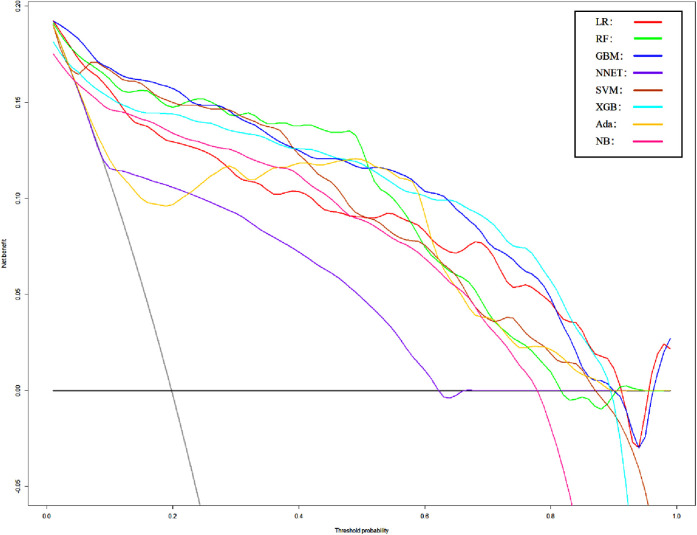
Decision curve analysis of Model2. LR, logistic regression; RF, random forest; GBM, gradient boosting machine; NNET, artificial neural network; SVM, support vector machine; XGB, eXtreme gradient boosting; Ada, adapting boosting; NB, naïve Bayes; Model2 was adjusted for GCS, glucose, sodium, chloride, SPO_2_, bicarbonate, temperature, white blood cell (WBC), heparin use, SOFA score, creatinine, bun, platelet, age, marital, trauma, lymphocytes, calcium, race, and cerebrovascular disease.

We use 10 features and 20 features to establish the traditional regression and ML models, respectively. In simplified Model 1, the logistic regression (LR), random forest (RF), gradient boosting machine (GBM), artificial neural network (NNET), support vector machine (SVM), eXtreme gradient boosting (XGB), adapting boosting (ADA), and naïve Bayes (NB) models obtained AUCs of 0.883, 0.949, 0.945, 0.888, 0.926, 0.925, 0.936, and 0.920, respectively (Table 2 and Figure 3). In Model 2, the LR, RF, GBM, NNET, SVM, XGB, ADA, and NB models obtained AUCs of 0.921, 0.958, 0.959, 0.801, 0.942, 0.950, 0.951, and 0.927, respectively (Table 3 and Figure 4). Through the Delong test, different models constructed by LR, NNET, and XGB algorithms are different (Table 4). Comparatively, RF-Model 1 had the highest predictive performance among these models. The decision curve is suitable for comparing the net benefits of the best model and alternative methods of clinical decision-making. Among the two different models, the net benefit of the model composed of the GBM algorithm is higher than that of other models, indicating that the model has a better effect in predicting the in-hospital mortality of SAH (Figures 5, 6).

**Table 2:** Prediction performance of Model1 in the testing set.

Model	Accuracy	Sensitivity	Specificity	PPV	NPV	AUC	95% CI
LR-Model1	0.902	0.904	0.889	0.982	0.577	0.883	0.874–0.925
RF-Nodel1	0.920	0.928	0.875	0.976	0.694	0.949	0.894–0.941
GBM-Model1	0.918	0.928	0.865	0.973	0.693	0.945	0.892–0.939
NNET-Model1	0.889	0.918	0.753	0.946	0.658	0.888	0.860–0.914
SVM-Model1	0.902	0.902	0.900	0.984	0.568	0.926	0.874–0.925
XGB-Model1	0.909	0.936	0.788	0.951	0.739	0.925	0.882–0.931
Ada-Model1	0.907	0.928	0.804	0.958	0.703	0.936	0.880–0.930
NB-Model1	0.895	0.926	0.755	0.944	0.694	0.920	0.866–0.919

*PPV, positive predictive values; NPV, negative predictive values; AUC, area under the curve; CI, confidence interval; LR, logistic regression; RF, random forest; GBM, gradient boosting machine; NNET, artificial neural network; SVM, support vector machine; XGB, eXtreme gradient boosting; Ada, adapting boosting; NB, naïve Bayes; Model1 was adjusted for GCS, glucose, sodium, chloride, SPO_2_, bicarbonate, temperature, white blood cell (WBC), heparin use, and SOFA score.*

**Table 3:** Prediction performance of Model2 in the testing set.

Model	Accuracy	Sensitivity	Specificity	PPV	NPV	AUC	95% CI
LR-Model2	0**.**893	0**.**910	0**.**800	0**.**962	0**.**613	0**.**921	0.864–0.917
RF-Model2	0**.**930	0**.**938	0**.**891	0**.**978	0**.**739	0**.**958	0.906–0.950
GBM-Model2	0**.**916	0**.**935	0**.**826	0**.**962	0**.**730	0**.**959	0.904–0.948
NNET-Model2	0**.**850	0**.**906	0**.**622	0**.**906	0**.**622	0**.**801	0.818–0.879
SVM-Model2	0**.**891	0**.**896	0**.**857	0**.**978	0**.**540	0**.**942	0.862–0.916
XGB-Model2	0**.**920	0**.**941	0**.**823	0**.**960	0**.**757	0**.**950	0.894–0.941
Ada-Model2	0**.**923	0**.**941	0**.**840	0**.**964	0**.**759	0**.**951	0.898–0.944
NB-Model2	0**.**891	0**.**924	0**.**745	0**.**942	0**.**685	0**.**927	0.862–0.916

*PPV, positive predictive values; NPV, negative predictive values; AUC, area under the curve; CI, confidence interval; LR, logistic regression; RF, random forest; GBM, gradient boosting machine; NNET, artificial neural network; SVM, support vector machine; XGB, eXtreme gradient boosting; Ada, adapting boosting; NB, naïve Bayes; Model2 was adjusted for GCS, glucose, sodium, chloride, SPO_2_, bicarbonate, temperature, white blood cell (WBC), heparin use, SOFA score, creatinine, bun, platelet, age, marital, trauma, lymphocytes, calcium, race, and cerebrovascular disease.*

**Table 4:** Delong test of models.

Model	Model	*P*	Model	Model	*P*
LR-Model1	LR-Model2	0.002	SVM-Model1	SVM-Model2	0.222
RF-Model1	RF-Model2	0.124	XGB-Model1	XGB-Model2	0.013
GBM-Model1	GBM-Model2	0.076	Ada-Model1	Ada-Model2	0.130
NNET-Model1	NNET-Model2	<0.001	NB-Model1	NB-Model2	0.377

*LR, logistic regression; RF, random forest; GBM, gradient boosting machine; NNET, artificial neural network; SVM, support vector machine; XGB, eXtreme gradient boosting; Ada, adapting boosting; NB, naïve Bayes; Model1 was adjusted for GCS, glucose, sodium, chloride, SPO_2_, bicarbonate, temperature, white blood cell (WBC), heparin use, and SOFA score. Model2 was adjusted for GCS, glucose, sodium, chloride, SPO_2_, bicarbonate, temperature, white blood cell (WBC), heparin use, SOFA score, creatinine, bun, platelet, age, marital, trauma, lymphocytes, calcium, race, and cerebrovascular disease.*

**Figure 7:**
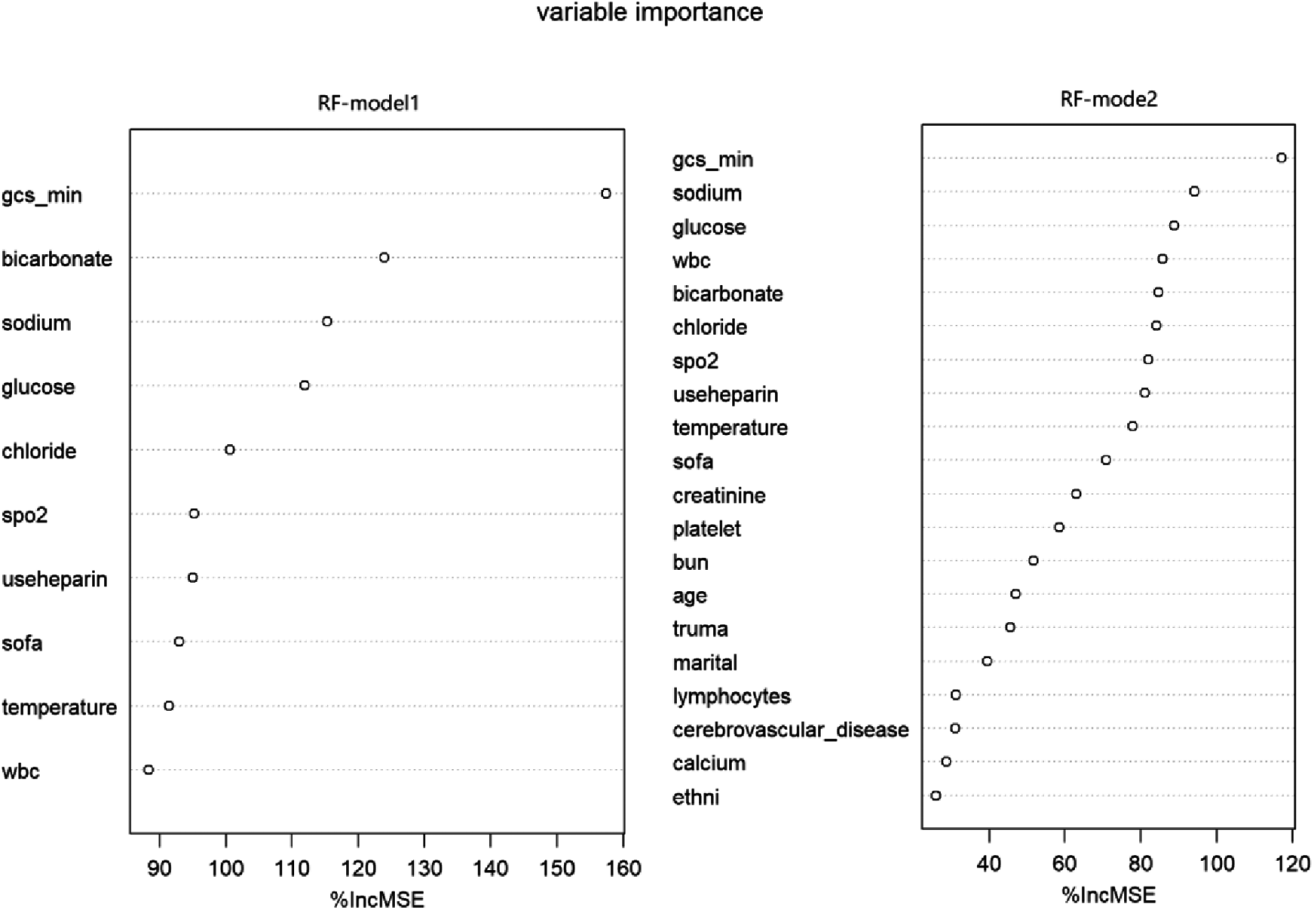
Variable importance in RF models. RF, random forest; Model1 was adjusted for GCS, glucose, sodium, chloride, SPO_2_, bicarbonate, temperature, white blood cell (WBC), heparin use, and SOFA score. Model2 was adjusted for GCS, glucose, sodium, chloride, SPO_2_, bicarbonate, temperature, white blood cell (WBC), heparin use, SOFA score, creatinine, bun, platelet, age, marital, trauma, lymphocytes, calcium, race, and cerebrovascular disease.

Through the importance ranking of the ML algorithm, the first 10 important characteristics of two different models of RF are consistent (Figure 7). Moreover, the importance of the GCS accounted for the highest proportion.

### Performance of Models in Subgroup (Non-Traumatic Subarachnoid Hemorrhage) Analysis

**Figure 8:**
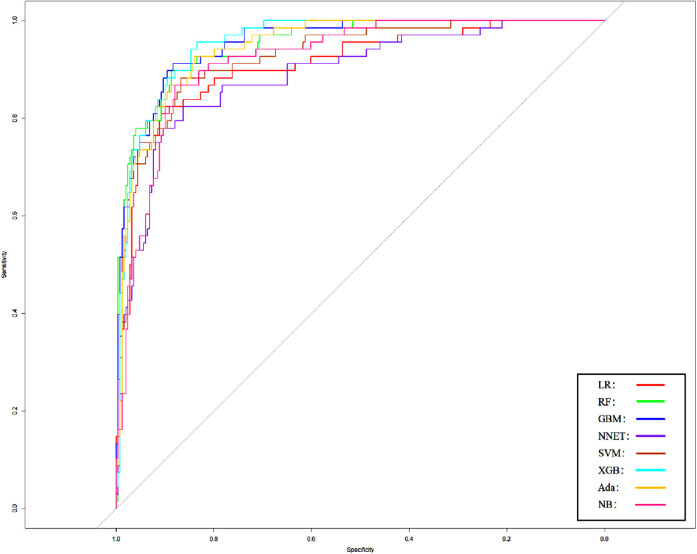
Area under the receiver operating characteristic curve of different models of non-traumatic subarachnoid hemorrhage in the validation cohort. LR, logistic regression; RF, random forest; GBM, gradient boosting machine; NNET, artificial neural network; SVM, support vector machine; XGB, eXtreme gradient boosting; Ada, adapting boosting; NB, naïve Bayes; the model was adjusted for GCS, glucose, sodium, chloride, SPO_2_, bicarbonate, temperature, white blood cell (WBC), heparin use, and SOFA score.

**Figure 9:**
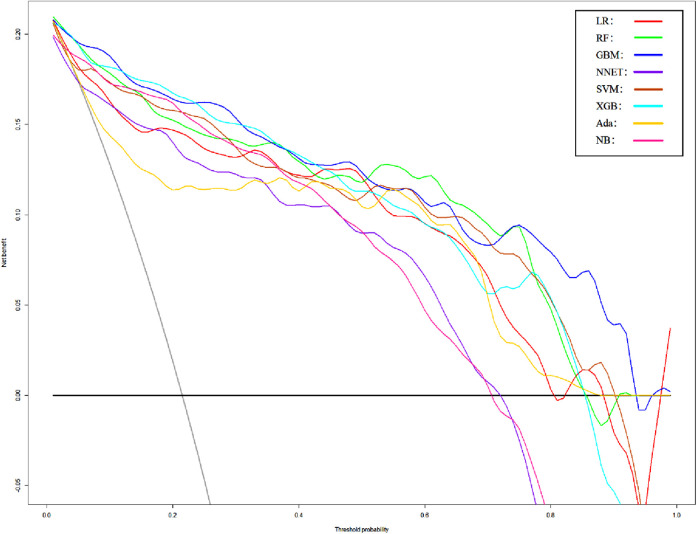
DCA curves of different models of non-traumatic subarachnoid hemorrhage. LR, logistic regression; RF, random forest; GBM, gradient boosting machine; NNET, artificial neural network; SVM, support vector machine; XGB, eXtreme gradient boosting; Ada, adapting boosting; NB, naïve Bayes; the model was adjusted for GCS, glucose, sodium, chloride, SPO_2_, bicarbonate, temperature, white blood cell (WBC), heparin use, and SOFA score.

In order to verify the prediction ability of the model in non-traumatic subarachnoid hemorrhage, we took the cases without definite trauma as a new research subgroup (Table 5) and divided them into a training set and a test set (ratio 70:30). After establishing the model with the simplified characteristic variables in the training set, the prediction ability was verified with the test set. Within the training set, the LR, RF, GBM, NNET, SVM, XGB, Ada, and NB models were established, and the testing set obtained AUCs of 0.909, 0.951, 0.955, 0.891, 0.929, 0.956, 0.947, and 0.921 (Table 6 and Figure 8). Among the eight models, GBM has the highest prediction performance and NNET has the worst generalization ability. As shown in Figure 9, the net benefit of the GBM model exceeded that of other ML models and LR regression models, indicating that the model has better performance in predicting the queue.

**Table 5:** Baseline characteristics of patients without traumatic subarachnoid hemorrhage.

	Survival (*n* = 741)	Dead in hospital (*n* = 240)	*P*-value
Baseline characteristics			
Age (year)	59 (50, 70)	69 (58, 80)	<0.001
Sex (female)	421 (57%)	131 (55%)	0.595
Race			
Black	1 (0%)	0 (0%)	<0.001
White	22 (3%)	12 (5%)	
Hispanic	63 (9%)	13 (5%)	
Asian	42 (6%)	8 (3%)	
Others	482 (65%)	106 (44%)	
Language			
English	662 (89%)	205 (85%)	0.126
Unknow	79 (11%)	35 (15%)	
Marital			
Single	193 (26%)	33 (14%)	<0.001
Married	365 (49%)	87 (36%)	
Divorced	66 (9%)	13 (5%)	
Widowed	49 (7%)	22 (9%)	
Unknow	68 (9%)	85 (35%)	
Weight	76.87 (65.5, 90.9)	73.2 (61, 90)	0.034
Coexisting disorders			
Myocardial infarction	52 (7%)	26 (11%)	0.066
Congestive heart failure	56 (8%)	31 (13%)	0.014
Peripheral vascular disease	54 (7%)	18 (8%)	0.913
Cerebrovascular disease	741 (100%)	240 (100%)	1.000
Dementia	14 (2%)	3 (1%)	0.494
Chronic pulmonary disease	102 (14%)	41 (17%)	0.212
Rheumatic disease	13 (2%)	4 (2%)	0.928
Peptic ulcer disease	4 (15)	3 (1%)	0.285
Diabetes	98 (13%)	44 (18%)	0.060
Paraplegia	110 (15%)	40 (17%)	0.499
Renal disease	40 (5%)	34 (14%)	<0.001
Malignant cancer	26 (4%)	14 (6%)	0.128
Severe liver disease	7 (1%)	9 (4%)	0.006
Metastatic solid tumor	10 (1%)	8 (3%)	0.062
AIDS	2 (0%)	2 (1%)	0.270
Vital signs (1st 24 h)			
Heart rate (min)	75.64 (69.04, 85.4)	80.3 (72.98, 92.05)	<0.001
Temperature (°C)	36.96 (36.77, 37.23)	36.95 (36.51, 37.44)	0.265
SBP (mmHg)	124(115, 134)	123(111, 134)	<0.001
DBP (mmHg)	65(58, 72)	62(55, 70)	<0.001
MBP (mmHg)	82(75, 88)	80(73, 88)	<0.001
Respiratory rate (min)	17 (16, 18)	19 (17, 21)	<0.001
SPO_2_	97.31 (96.03, 98.77)	97.92 (95.59, 99.29)	0.104
Laboratory			
WBC	10.1 (8.37, 12.03)	13.05 (10.35, 16.2)	<0.001
Hematocrit	34.43 (30.83, 38.3)	33.55 (29.34, 37.53)	0.062
Hemoglobin	11.53 (10.2, 12.9)	11.1 (9.41, 12.7)	0.007
Mch	30.47 (29.43, 31.66)	30.5 (29.1, 31.72)	0.775
Mchc	33.4 (32.58, 34.28)	33.1 (32.06, 33.91)	<0.001
Mcv	91 (87, 94)	92 (88, 95)	0.018
RBC	3.81 ± 0.61	3.72 ± 0.75	0.088
Rdw	13.57 (12.99, 14.54)	14.35 (13.47, 16.08)	<0.001
Platelet	243.09 (199.5, 306.2)	205.56 (147.31, 261.82)	<0.001
Neutrophils	78.2 (73.2, 84.6)	79.88 (77.59, 86.4)	0.003
Lymphocytes	13.93 (9.4, 17.3)	12.45 (6.53, 14.2)	<0.001
Monocytes	5.4 (3.6, 6.3)	5.86 (4, 6)	0.169
Eosinophils	0.8 (0.3, 1.3)	0.7 (0.2, 1.1)	<0.001
Basophils	0.35 (0.2, 0.48)	0.33 (0.2, 0.36)	<0.001
Bicarbonate	24.15 (22.76, 25.86)	22.64 (19.8, 24.63)	<0.001
Bun	13.33 (10.5, 18)	19.86 (14.63, 30.21)	<0.001
Calcium	8.67 (8.42, 8.96)	8.45 (8.09, 8.67)	<0.001
Chloride	104 (102, 106.32)	106 (103.27, 111.58)	<0.001
Creatinine	0.71 (0.58, 0.87)	0.95 (0.69, 1.42)	<0.001
Glucose	119.21 (108.71, 134.28)	153.32 (131.89, 181.5)	<0.001
Sodium	139.3 (137.44, 141.33)	141.17 (139.08, 145.62)	<0.001
Potassium	3.89 (3.74, 4.1)	3.99 (3.76, 4.28)	0.003
INR	1.1 (1.04, 1.19)	1.17 (1.1, 1.35)	<0.001
PT	12.28 (11.5, 13.2)	13.04 (11.9, 14.74)	<0.001
APTT	28.48 (26.29, 31.48)	29.04 (26.29, 35.11)	0.033
NLR	7.39(4.28, 9.05)	10.29(5.46, 13.21)	<0.001
Therapy			
Heparin	639 (86)	104 (43)	<0.001
Antibiotic	435 (59)	156 (65)	0.098
Scoring system			
SOFA	3 (2, 5)	6 (4, 8.25)	<0.001
GCS	13 (9, 14)	8 (3, 15)	0.002

*AIDS, acquired immunodeficiency syndrome; SBP, systolic blood pressure; DBP, diastolic blood pressure; MBP, mean blood pressure; Mch, mean corpuscular hemoglobin; Mchc, mean corpuscular hemoglobin concentration; Mcv, mean corpuscular volume; RBC, red blood cell; Rdw, red blood cell volume distribution width; SPO_2_, oxygen saturation; GCS, Glasgow coma score; SOFA, sepsis-related organ failure assessment; and NLR, neutrophil-to-lymphocyte ratio.*

**Table 6:** The prediction performance of the non-traumatic subarachnoid hemorrhage prediction model in the test set.

Model	Accuracy	Sensitivity	Specificity	PPV	NPV	AUC	95%CI
LR-model	0.905	0.926	0.817	0.956	0.720	0.909	0.867–0.935
RF-model	0.902	0.943	0.760	0.931	0.794	0.951	0.864–0.932
GBM-model	0.908	0.933	0.809	0.952	0.750	0.955	0.871–0.938
NNET-model	0.873	0.930	0.689	0.907	0.750	0.891	0.832–0.910
SVM-model	0.892	0.921	0.774	0.943	0.706	0.929	0.853–0.924
XGB-model	0.899	0.943	0.750	0.927	0.794	0.956	0.860–0.930
Ada-model	0.892	0.938	0.736	0.923	0.779	0.947	0.853–0.924
NB-model	0.877	0.934	0.693	0.907	0.764	0.921	0.835–0.911

*PPV, positive predictive values; NPV, negative predictive values; AUC, area under the curve; CI, confidence interval; LR, logistic regression; RF, random forest; GBM, gradient boosting machine; NNET, artificial neural network; SVM, support vector machine; XGB, eXtreme gradient boosting; Ada, adapting boosting; NB, naïve Bayes; Model1 was adjusted for GCS, glucose, sodium, chloride, SPO_2_, bicarbonate, temperature, white blood cell (WBC), heparin use, and SOFA score*.

## Discussion

Subarachnoid hemorrhage (SAH) has a high mortality and disability rate, and many complications may occur after the onset, while most of the current studies have used a single feature for prognosis research, ignoring the adverse outcomes caused by other factors. Recently, a large number of studies have reported that peripheral blood, biochemical, and other conventional indicators are associated with the prognosis of subarachnoid hemorrhage, so we used the indicators commonly found in the mimic database for model building.

In this study, we use RFE to screen important features. After simplifying the model, we use the traditional logistic regression and ML algorithm for modeling. There is basically no significant difference in the prediction ability between these simplified models and the best models. At the same time, the simplified models can reduce the phenomenon of overfitting and are more suitable for clinical use to reduce unnecessary workloads. In subgroup analysis, the model established with the same characteristics has higher AUCs, which also proves that the model has a better ability to predict the prognosis of patients with non-traumatic subarachnoid hemorrhage.

From the study, we found a larger association of mortality with patients’ electrolyte levels, glucose levels, and whether they used heparin in addition to the traditional GCS. In addition, the SOFA score also history a significant mortality factor, and this score mainly describes indicators of impairment in multiple organ functions (10) (respiratory, neurological, cardiovascular, hepatic, coagulation, and renal). The underlying mechanism may be caused by the patient’s past medical history leading to organ failure or by coagulopathy due to bleeding.

Impaired consciousness occurs in some patients after SAH. GCS is assessed by the ability to eye opening, best verbal response, and best motor response, can easily and rapidly assess the state of consciousness of a patient, and to identify development of complications and the potential degree of ultimate recovery (11). Meanwhile, in our study, glucose level served as an important factor in the prediction of death. Pppacena et al. found that higher blood glucose was associated with higher mortality after SAH (12). Meanwhile, a higher rate of glycemic variability was also associated with prognosis after SAH (13).

Recently, the neutrophil to lymphocyte ratio (NLR) was reported by most literature studies to have a correlation with the prognosis of SAH (14), so we also calculated NLR as a feature. In univariate analysis, there was a clear difference between the two groups, and after filtering by ML algorithms, NLR failed to be included in the model as a better feature, perhaps because of inconsistent outcomes across studies. The higher importance of leukocytes at the same time is consistent with the finding by Srinivasan et al. and Chamling et al. that early elevation of peripheral leukocytes is associated with the occurrence of DCI and poor functional outcomes (15).

Sodium and chloride are important components of electrolytes in humans, and 36% of SAH patients present with hyponatremia after the onset, mainly as a result of cerebral salt-wasting syndrome (CSWS) and syndrome of inappropriate antidiuretic hormone secretion (SIADH). Vrsajkov et al. and Saramma et al. found better outcomes in patients who did not develop hyponatremia during ICU treatment (16, 17). Hyponatremia has also been reported to be associated with an increased risk of vasospasm. This may be the main reason for the poor prognosis of patients (18).

Low bicarbonate concentrations occur in patients with severe acute illness. Although the current mechanism is unknown, increased systemic vascular resistance can occur after SAH, leading to transient lactic acidosis with the formation of neurogenic pulmonary edema, resulting in poor patient outcomes reported in a case study (19), Satoh et al. found that patients presenting with neurogenic pulmonary edema had lower bicarbonate concentrations (20). In addition, Stephan et al. found that one in five patients had abnormally low bicarbonate levels on admission and a poor prognosis (21).

Our study found that the use of heparin in SAH patients was able to improve outcomes, which was consistent with the findings of Post et al. (22) that the use of heparin was able to reduce mortality after SAH. The concomitant use of low-dose heparin may reduce the risk of thrombosis and reduce the poor prognosis resulting from thrombus shedding (23, 24).

In summary, the characteristic factors screened by RFE in our study were all investigated in SAH; meanwhile, they were all correlated with prognosis. The strength of this study is that the method of ML was used to combine the relevant factors to predict the mortality of SAH, while feature acquisition was simple and able to be acquired within a smaller hospital. Patients with SAH are sicker, and early and accurate prediction of mortality is able to provide clinicians with more time to adjust the corresponding treatment options, while, at the same time, in clinical work, further treatment should be given to the related diseases. In addition, the validation set was adopted in this study to verify the reliability of the model so that it had better reliability. Finally, most of the data in this study come from publicly available databases, and their data have good reliability.

Our study has limitations, which are similar to most studies related to public databases. First, the MIMIC database cannot provide the relevant imaging examination of cases. Therefore, we cannot perform an M-Fisher score on patients to establish a model nor can we evaluate whether patients have obvious trauma information and the nature of aneurysms. Second, as a public database, the MIMIC database may cause data errors due to the errors of researchers or the database itself when extracting data. In addition, there is the possibility of SAH error classification. In order to reduce the deviation caused by inaccurate code, we adopt the extensively used ICD-9 and -10 codes. Third, as with all potential retrospective studies, there are unmeasured confounding factors that lead to selection bias. Finally, although our study explored the mortality of SAH in the intensive care unit, other results, such as long-term prognosis and complications, also need further study.

## Conclusion

This study suggests that some important features may be related to the prognosis after SAH. The ML model deals with a large number of variables and then distinguishes patients who die in hospitals to promote the implementation of timely and effective treatment. In the future, further verification of its clinical application value will be necessary.

## Data Availability

Publicly available data sets were analyzed in this study. This data can be found here: https://mimic.mit.edu/.

## References

[1] Go AS Mozaffarian D Roger VL Benjamin EJ Berry JD Blaha MJ Heart disease and stroke statistics—2014 update: a report from the American Heart Association. Circulation. (2014) 129(3):e28–292. 10.1161/01.cir.0000441139.02102.80 24352519 PMC5408159

[2] Lovelock CE Rinkel GJE Rothwell PM. Time trends in outcome of subarachnoid hemorrhage: population-based study and systematic review. Neurology. (2010) 74(19):1494–501. 10.1212/WNL.0b013e3181dd42b3 20375310 PMC2875923

[3] Muehlschlegel S. Subarachnoid hemorrhage. Contin Lifelong Learn Neurol. (2018) 24(6):1623–57. 10.1212/CON.0000000000000679 30516599

[4] Beam AL Kohane IS. Big data and machine learning in health care. JAMA. (2018) 319(13):1317. 10.1001/jama.2017.18391 29532063

[5] Zhang Z Ho KM Hong Y. Machine learning for the prediction of volume responsiveness in patients with oliguric acute kidney injury in critical care. Crit Care. (2019) 23(1):112. 10.1186/s13054-019-2411-z 30961662 PMC6454725

[6] Zhang Z. Predictive analytics in the era of big data: opportunities and challenges. Ann Transl Med. (2020) 8(4):68–8. 10.21037/atm.2019.10.97 32175361 PMC7049053

[7] Goldberger AL Amaral LA Glass L Hausdorff JM Ivanov PC Mark RG PhysioBank, PhysioToolkit, and PhysioNet: components of a new research resource for complex physiologic signals. Circulation. (2000) 101:e215-20. 10.1161/01.CIR.101.23.e215 10851218

[8] Sanz H Valim C Vegas E Oller JM Reverter F. SVM-RFE: selection and visualization of the most relevant features through non-linear kernels. BMC Bioinformatics. (2018) 19(1):432. 10.1186/s12859-018-2451-4 30453885 PMC6245920

[9] Chen Q Meng Z Liu X Jin Q Su R. Decision variants for the automatic determination of optimal feature subset in RF-RFE. Genes. (2018) 9(6):301. 10.3390/genes9060301 PMC6027449 29914084

[10] Lambden S Laterre PF Levy MM Francois B. The SOFA score—development, utility and challenges of accurate assessment in clinical trials. Crit Care. (2019) 23(1):374. 10.1186/s13054-019-2663-7 31775846 PMC6880479

[11] Middleton PM. Practical use of the Glasgow Coma Scale: a comprehensive narrative review of GCS methodology. Australas Emerg Nurs J. (2012) 15(3):170–83. 10.1016/j.aenj.2012.06.002 22947690

[12] Pappacena S Bailey M Cabrini L Landoni G Udy A Pilcher DV Early dysglycemia and mortality in traumatic brain injury and subarachnoid hemorrhage. Minerva Anestesiol. (2019) 85(8):830–9. 10.23736/S0375-9393.19.13307-X 30735020

[13] Okazaki T Hifumi T Kawakita K Shishido H Ogawa D Okauchi M Blood glucose variability: a strong independent predictor of neurological outcomes in aneurysmal subarachnoid hemorrhage. J Intensive Care Med. (2018) 33(3):189–95. 10.1177/0885066616669328 27630011

[14] Al-Mufti F Amuluru K Damodara N Dodson V Roh D Agarwal S Admission neutrophil–lymphocyte ratio predicts delayed cerebral ischemia following aneurysmal subarachnoid hemorrhage. J NeuroInterventional Surg. (2019) 11(11):1135–40. 10.1136/neurintsurg-2019-014759 30979846

[15] Srinivasan A Aggarwal A Gaudihalli S Mohanty M Dhandapani M Singh H Impact of early leukocytosis and elevated high-sensitivity C-reactive protein on delayed cerebral ischemia and neurologic outcome after subarachnoid hemorrhage. World Neurosurg. (2016) 90:91–5. 10.1016/j.wneu.2016.02.049 26898490

[16] Vladimir V Gordana J Snezana S Arsen U Pantic VJ. Clinical and predictive significance of hyponatremia after aneurysmal subarachnoid hemorrhage. Balk Med J. (2012). 10.5152/balkanmedj.2012.037 PMC4115839 25207008

[17] Saramma PP Girish Menon P Srivastava A Sankara Sarma P. Hyponatremia after aneurysmal subarachnoid hemorrhage: implications and outcomes. J Neurosci Rural Pract. (2013) 04(01):24–8. 10.4103/0976-3147.105605 PMC3579037 23546343

[18] Maimaitili A Maimaitili M Rexidan A Lu J Ajimu K Cheng X Pituitary hormone level changes and hypxonatremia in aneurysmal subarachnoid hemorrhage. Exp Ther Med. (2013) 5(6):1657–62. 10.3892/etm.2013.1068 23837049 PMC3702695

[19] Mayer SA Fink ME Homma S Sherman D LiMandri G Lennihan L Cardiac injury associated with neurogenic pulmonary edema following subarachnoid hemorrhage. Neurology. (1994) 44(5):815–5. 10.1212/WNL.44.5.815 8190280

[20] Satoh E Tagami T Watanabe A Matsumoto G Suzuki G Onda H Association between serum lactate levels and early neurogenic pulmonary edema after nontraumatic subarachnoid hemorrhage. J Nippon Med Sch. (2014) 81(5):305–12. 10.1272/jnms.81.305 25391699

[21] Claassen J Vu A Kreiter KT Kowalski RG Du EY Ostapkovich N Effect of acute physiologic derangements on outcome after subarachnoid hemorrhage*. Crit Care Med. (2004) 32(3):832–8. 10.1097/01.CCM.0000114830.48833.8A 15090970

[22] Post R Zijlstra I Berg Rvd Coert BA Verbaan D Vandertop WP. High-dose nadroparin following endovascular aneurysm treatment benefits outcome after aneurysmal subarachnoid hemorrhage. Neurosurgery. (2018) 83(2):281–7. 10.1093/neuros/nyx381 28945859

[23] Hantsche A Wilhelmy F Kasper J Wende T Hamerla G Rasche S Early prophylactic anticoagulation after subarachnoid hemorrhage decreases systemic ischemia and improves outcome. Clin Neurol Neurosurg. (2021) 207:106809. 10.1016/j.clineuro.2021.106809 34274657

[24] Kunz M Siller S Nell C Schniepp R Dorn F Huge V Low-dose versus therapeutic range intravenous unfractionated heparin prophylaxis in the treatment of patients with severe aneurysmal subarachnoid hemorrhage after aneurysm occlusion. World Neurosurg. (2018) 117:e705–11. 10.1016/j.wneu.2018.06.118 29959066

